# Genital lichen sclerosus and melanoma; a systematic review

**DOI:** 10.1002/ski2.198

**Published:** 2022-12-11

**Authors:** Sharmaine J. Y. Sim, Katherine Dear, Evanthia Mastoraki, Mariel James, Aiman Haider, Peter Ellery, Alex Freeman, Hussain M. Alnajjar, Asif Muneer, Richard Watchorn, Georgios Kravvas, Christopher B. Bunker

**Affiliations:** ^1^ University College London Medical School London UK; ^2^ Department of Dermatology University College London Hospitals NHS Foundation Trust London UK; ^3^ Department of Histopathology University College London Hospitals NHS Foundation Trust London UK; ^4^ Department of Urology University College London Hospitals NHS Foundation Trust London UK

## Abstract

**Background:**

Lichen sclerosus (LSc) is a chronic, inflammatory, destructive skin disease with a predilection for the genitalia (GLSc). An association with vulval (Vu) and penile (Pe) squamous carcinoma (SCC) is now well established but melanoma (MM) has only rarely been reported complicating GLSc.

**Methods:**

We have performed a systematic literature review of GLSc in patients with genital melanoma (GMM). Only articles that mentioned both GMM and LSc affecting either the penis or vulva were included.

**Results:**

Twelve studies with a total of 20 patients were included. Our review shows that an association of GLSc with GMM has been more frequently reported in women and female children than men viz, 17 cases compared with three. It is notable that five of the cases (27.8%) concerned female children aged under twelve.

**Discussion:**

These data suggest a rare association between GLSc and GMM. If proven, there arise intriguing questions about pathogenesis and consequences for counselling of patients and follow‐up.

## Graphical abstract

A systematic literature review on the association between genital lichen sclerosus (GLSc) and genital melanoma (GMM).

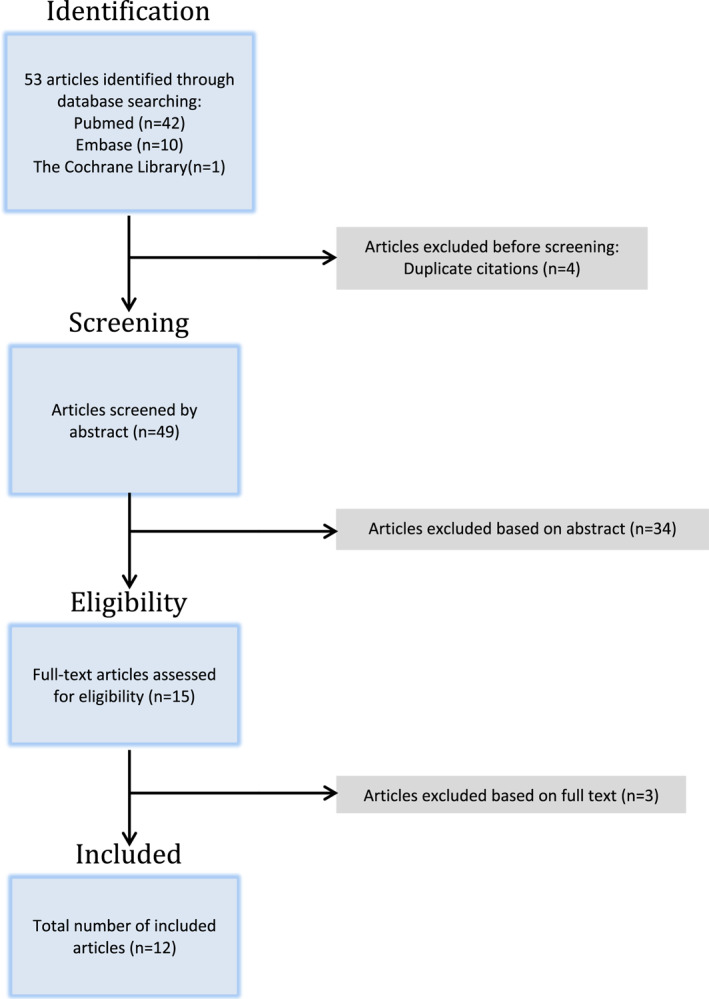

**What's already known about this topic?**

- Lichen sclerosus (LSc) is a chronic, inflammatory, destructive, and scarring skin disease with a predilection for the genitalia. Even though genital (G) LSc is thought to be a risk factor for the development of genital squamous cell carcinoma (GSCC), very little is known on the association between GLSc and genital melanoma (GMM).

**What does this study add?**

- The association of GLSc with GMM has been more frequently reported in women and children than men. There is no evidence in the published literature that links genital human papillomavirus (HPV) and GMM.

## 1 INTRODUCTION

Lichen sclerosus (LSc) is a chronic, inflammatory, destructive skin disease with a predilection for the genitalia (GLSc). An association with vulval (Vu) and penile (Pe) squamous cell carcinoma (SCC) is now well established.^1^, ^2^, ^3^ Reports of an association with genital (G) melanoma (MM) have appeared in the literature. We have previously published three cases of penile (Pe) MM complicating male (M) GLSc and recently encountered another case in our multidisciplinary practice.^4^ We have therefore undertaken a literature review in an attempt to shed more light on this topic.

## 2 METHODS

We performed a literature search on 8 November 2021 on PubMed (Medline), Cochrane Library, and Embase using prespecified search criteria.

The search terms used were (‘penis’ OR ‘penile’ OR ‘genitalia’ OR ‘genital’ OR ‘vulva’ OR ‘vulvar’ OR ‘vulval’) AND ‘melanoma’ AND (‘lichen sclerosus’ OR ‘lichen’).

Titles and abstracts were screened by two reviewers for relevance. Any discrepancies in findings were resolved by a third, senior reviewer.

We included published articles with GMM amongst patients with known GLSc, in any language, country and publication date. Articles that mentioned GMM alone, without reference to GLSc, were excluded. Likewise, articles that mentioned GLSc alone, without reference to GMM were excluded. Only articles that mentioned both GMM and GLSc on either the penis or vulva were included.

## 3 RESULTS

**FIGURE 1:**
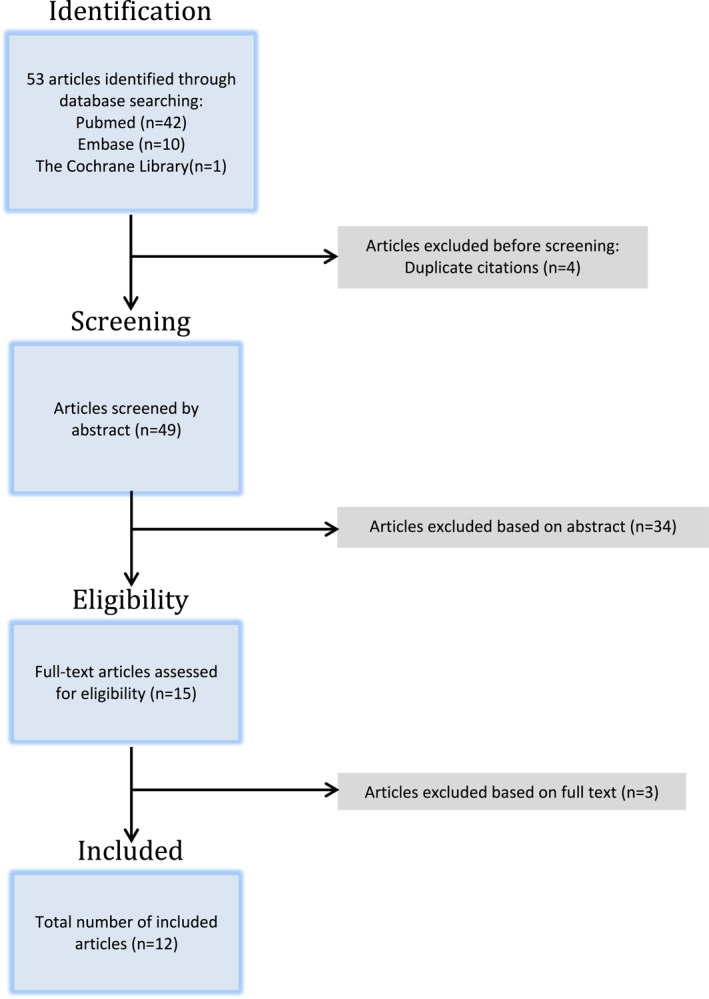
PRISMA flow diagram of the article selection process.

A total of 53 articles were identified through our search across all three databases (Embase = 10, Cochrane = 1, PubMed = 42). After excluding four duplicates, the remaining 49 articles were screened through their abstracts and 34 articles were excluded. The remaining 15 articles were then assessed for relevance based on their full text and a total of 12 studies were deemed eligible for inclusion (Figure 1).

Twenty patients were reported to have had GLSc associated with MM. There were five cases of female children, 12 cases of adult women, and three cases of adult men.^4^, ^5^, ^6^, ^7^, ^8^, ^9^, ^10^, ^11^, ^12^, ^13^, ^14^, ^15^ We strongly suspect however, that the publications by Carlson et al. and Rohwedder et al., both describe the same patient.

Amongst the five cases of female children, there was a 10‐year‐old with two separate sites of GLSc associated with MM, both found on the left vulva.^7^

The average ages of the female children, adult women, and adult men were 10, 74, and 40 years, respectively.

The mean Breslow thicknesses of the MMs for female children, adult women, and adult men were 0.55, 6.18, and 0.58 mm, respectively. In three patients this information was not available.

Data on Clark Levels were available in 11 cases with a calculated median value of 4.

Data on lymph node biopsies were available in six patients (five adult women and one female child); only in two (a 10‐year‐old female with two separate melanomas of the left vulva, and a 59‐year‐old female) were the lymph node biopsies found to be positive.^7^, ^8^

Only eight patients had information on the subtype of their melanomas. Of these, three were nodular, three were lentiginous, and two were superficial spreading.

A summary of the patient demographics and clinical findings is presented in Table 1.

**TABLE 1:** A summary of patient demographics and clinical findings

Author	Year	No. of patients	Age in years	Gender	Subtype of melanoma	Clark level	Breslow thickness (in mm)	Lymph node biopsy
Ulmer et al.	1996	1	60	F	Superficial spreading	4	1.4	N/A
Egan et al.	1997	2	9	F	N/A	N/A	N/A	N/A
			11	F	N/A	N/A	0.47	N/A
Carlson et al.^a^	2002	1	83	F	Nodular	4	2.7	Neg.
Rohwedder et al.^a^	2002	1	83	F	N/A	4	2.7	N/A
Hassanein et al.	2004	1	10	F	Lentiginous	2	0.44	Neg.
Rosamilia et al.	2006	1	10	F	N/A	N/A	0.36 & 1	Pos. & pos.
Rohwedder et al.	2007	2	76	F	Lentiginous	4	2	N/A
			85	F	Nodular	4	7	N/A
De Simone et al.	2008	1	N/A	F	Superficial spreading	N/A	N/A	N/A
Heinzelmann et al.	2014	3	69	F	N/A	N/A	1	Neg.
			81	F	N/A	N/A	N/A	N/A
			84	F	N/A	N/A	3.5	Neg.
Spina et al.	2016	1	11	F	N/A	3	0.5	N/A
Turnbull et al.	2016	3	30	M	Lentiginous	N/A	0.1	N/A
			40	M	N/A	N/A	0.44	N/A
			50	M	Nodular	5	1.2	N/A
Hieta et al.	2018	3	59	F	N/A	4	10	Pos.
			69	F	N/A	5	19	N/A
			79	F	N/A	4	9	N/A

^a^ Publications that are suspected to have reported on the same patient.

## 4 DISCUSSION

LSc is a chronic, inflammatory, destructive, and scarring skin disease with a predilection for the genitalia. Female (F) GLSc most commonly affects postmenopausal women with the prevalence estimated to be 1 in 30 older women. FGLSc is also held to be 6‐10‐fold more common than MGLSc.^1^ However, the exact incidence and prevalence of GLSC is unknown in males; this is also the case with children. In males the incidence appears trimodal—children, young males, and older males. MGLSc may be much more frequent than is generally supposed in early childhood (being diagnosed histologically in 14%–95% of prepuces removed for phimosis).^2^, ^3^

FGLSc most commonly affects the genitocrural folds, the inner aspects of labia majora, labia minora, clitoris and clitoral hood. FGLSc is also commonly associated with perianal LSc in a characteristic figure‐of‐8 distribution.^1^, ^2^ In MGLSc the disease affects the balanopreputial epithelium and can involve the urethra (which is rare in women). When left undiagnosed and inadequately treated, GLSc can cause dyspareunia, urinary difficulties, scarring, and irreversible architectural and pigmentary changes. It is also associated with a risk of progression to SCC as discussed below.^1^, ^2^, ^3^

The pathogenesis of GLSc is controversial, being most likely multifactorial; important postulated hypotheses are chronic irritation of epithelium by urine, dysbiosis, and autoimmunity.^16^, ^17^, ^18^

In childhood LSc the risks of vulvar and penile malignancy are unclear. However, in adult women with FGLSc the incidence of VuSCC is thought to be about 4%–12%.^1^, ^11^ In MGLSc the published rates vary between 0% and 12.5% (with the lower figures from our group, testifying to early, accurate diagnosis and effective definitive treatment).^19^, ^20^, ^21^, ^22^ GLSc has also been reported in association with other neoplasms, including Merkel cell carcinoma, spindle cell carcinoma, vulvar malignant pleomorphic adenoma and basal cell carcinoma, as well as with benign nevi.^9^, ^23^, ^24^, ^25^, ^26^

VuSCC emanating from FGLSc has been postulated to be a consequence of the molecularly altered epithelium created by chronic inflammation and abnormal cytokine and growth‐factor production.^11^, ^27^ In men, the carcinogenic pathway has not previously generated much comment or research but is an active area of investigation by our group.

Mucosal MM is rare, representing less than 2% of all MM.^28^ Vaginal (Va) and VuMM constitute between 1% and 3% of MM diagnosed in women, while PeMM accounts for 1.4% of primary penile cancers, and less than 0.1% of primary MM.^29^, ^30^, ^31^

Vu/Va MM typically occurs in post‐menopausal women and has an estimated incidence of 0.10/100 000 women/year.^8^, ^15^, ^32^ It is even rarer in children, with the incidence of childhood MM cited as being between 0.8 and 8 per million.^8^, ^9^, ^33^, ^34^

PeMM seems to be even rarer with only approximately 200 cases reported in the literature.^31^ Our review shows that the association of GLSc with MM has been more frequently reported in women and children than men that is, 17 cases compared with three.^4^, ^5^, ^6^, ^7^, ^8^, ^9^, ^10^, ^11^, ^12^, ^13^, ^14^, ^15^ Hence, we plan to review all the cases of PeMM and VuMM seen in our multidisciplinary practice in order to further investigate this.

In most cases MM arises in sun‐exposed skin.^35^ Mucosal MM develops from the epithelium of the oropharynx, gastrointestinal and genital tracts.^36^ While UV radiation is important in the pathogenesis of cutaneous melanoma, the pathogenesis of mucosal MM is unclear.^4^ Mucosal and cutaneous MM have genetic, histological, and clinical differences and should therefore be contemplated as distinct entities.^8^, ^9^, ^10^, ^12^, ^30^ VuMM presents at a more advanced stage and has a much poorer prognosis than cutaneous MM (47% vs. 80% 5‐year survival).^12^, ^13^, ^15^, ^30^, ^32^, ^37^, ^38^, ^39^ This is thought to be due to the essential biological aggressiveness of the neoplasm, the lack of early symptoms, a thin underlying dermis, delayed diagnosis, and less radical surgical therapy.^15^, ^40^, ^41^ PeMM is similarly considered to be an aggressive malignancy.^31^

Even though ultraviolet radiation is an important aetiological factor for cutaneous MM, this cannot be a factor in genital melanomagenesis.^15^ Therefore, the mutagenic agents involved in Vu and PeMM are likely to involve local stressors such as chronic inflammation, genital melanosis, viral infections (such as HPV), irritant agents, and perhaps genetic susceptibility.^9^, ^12^, ^13^, ^15^, ^42^ In the paper by Hieta et al., it was found that 12% (30/249) of patients with FGLSc had VuSCCs, but only 0.01% (3/249) had VuMM.^11^ But, they calculated that the risk of VuMM among patients with FGLSc was 341 times higher than the risk in women with no GLSc.^11^

The higher incidence of vulvar SCC among patients with LSc has been postulated to be a result of the molecularly altered epithelium generated by the effects of chronic inflammation with abnormal cytokine and growth‐factor production.^11^, ^27^

While melanocytic nevi can be found coinciding with GLSc, in two cases of MM associated with GLSc the histologic changes of LSc were found at the periphery of the MM but not immediately adjacent to or underlying the MM.^9^, ^12^, ^43^ LSc always vanished beneath the invasive melanoma, where dermal hyalinization was replaced by fibrosis.^10^

HPV has been detected in GLSc and it has been postulated that the inflammatory environment of LSc may generate a pro‐oxidative environment that, in conjunction with oncogenic HPV, produces a field effect that initiates and facilitates development of MM.^9^, ^12^, ^44^, ^45^, ^46^, ^47^, ^48^, ^49^, ^50^, ^51^, ^52^ Keratinocytes and melanocytes in the epithelium affected by LSc may be more vulnerable to oxidative and genotoxic insults because the lack of melanin would normally confer some protection.^53^, ^54^, ^55^, ^56^

However, although HPV has also been detected in both primary melanoma and acquired dysplastic melanocytic naevi, the association between HPV and MM is still unclear. High‐risk HPV was not identified in association with MM in neither of the studies by Rohwedder et al. and Ueda et al.^12^, ^13^, ^57^, ^58^

It remains the case that an underlying mechanism to explain the association between GLSc and GMM has yet to be elucidated. However, a possible hypothesis is that chronic melanocytic distress is created by a pro‐oxidative environment that increases mutagenesis and attenuates or disrupts local protective mechanisms.^9^

The limited number of reports on the association of GLSc with GMM may be partly due to lack of recognition of the link between the two conditions, as well as under‐reporting.^10^, ^43^, ^59^ The striking histological features of MM are likely to overshadow the more subtle features of MGLSc. In addition, melanosis in GLSc is common, and melanocytic proliferations in GLSc can be challenging to interpret, with benign melanocytic nevi occurring in GLSc sometimes histologically resembling malignant melanoma.^5^, ^6^, ^9^, ^43^, ^60^, ^61^, ^62^, ^63^, ^64^, ^65^, ^66^, ^67^

Despite the small number of reports, it is likely that the colocalization between GMM and GLSc is much more common than originally thought. Clinicians need to be aware of this important association in order to counsel and follow‐up patients accordingly.

## CONFLICT OF INTEREST

The authors have no conflicts of interest to disclose.

## AUTHOR CONTRIBUTIONS

**Sharmaine Sim**: Conceptualization (Equal); Formal analysis (Equal); Methodology (Equal); Project administration (Equal); Writing – original draft (Equal); Writing – review & editing (Equal). **Katherine Dear**: Conceptualization (Equal); Formal analysis (Equal); Methodology (Equal); Project administration (Equal); Writing – original draft (Equal); Writing – review & editing (Equal). **Evanthia Mastoraki**: Conceptualization (Equal); Formal analysis (Equal); Methodology (Equal); Project administration (Equal); Writing – original draft (Equal); Writing – review & editing (Equal). **Mariel James**: Conceptualization (Equal); Formal analysis (Equal); Methodology (Equal); Project administration (Equal); Writing – original draft (Equal); Writing – review & editing (Equal). **Aiman Haider**: Conceptualization (Equal); Formal analysis (Equal); Methodology (Equal); Project administration (Equal); Writing – original draft (Equal); Writing – review & editing (Equal). **Peter Ellery**: Conceptualization (Equal); Formal analysis (Equal); Methodology (Equal); Project administration (Equal); Writing – original draft (Equal); Writing – review & editing (Equal). **Alex Freeman**: Conceptualization (Equal); Formal analysis (Equal); Methodology (Equal); Project administration (Equal); Writing – original draft (Equal); Writing – review & editing (Equal). **Hussain M. Alnajjar**: Conceptualization (Equal); Formal analysis (Equal); Methodology (Equal); Project administration (Equal); Writing – original draft (Equal); Writing – review & editing (Equal). **Asif Muneer**: Conceptualization (Equal); Formal analysis (Equal); Methodology (Equal); Project administration (Equal); Writing – original draft (Equal); Writing – review & editing (Equal). **Richard Watchorn**: Conceptualization (Equal); Formal analysis (Equal); Methodology (Equal); Project administration (Equal); Writing – original draft (Equal); Writing – review & editing (Equal). **Georgios Kravvas**: Conceptualization (Equal); Formal analysis (Equal); Methodology (Equal); Project administration (Equal); Resources (Lead); Supervision (Lead); Writing – original draft (Equal); Writing – review & editing (Equal). **Christopher B. Bunker**: Conceptualization (Equal); Formal analysis (Equal); Methodology (Equal); Project administration (Equal); Resources (Lead); Supervision (Lead); Writing – original draft (Equal); Writing – review & editing (Equal).

## ETHICS STATEMENT

Not applicable.

## Data Availability

Data sharing is not applicable to this article as no new data were created or analyzed in this study.
